# A consensus map of rapeseed (*Brassica napus* L.) based on diversity array technology markers: applications in genetic dissection of qualitative and quantitative traits

**DOI:** 10.1186/1471-2164-14-277

**Published:** 2013-04-23

**Authors:** Harsh Raman, Rosy Raman, Andrzej Kilian, Frank Detering, Yan Long, David Edwards, Isobel AP Parkin, Andrew G Sharpe, Matthew N Nelson, Nick Larkan, Jun Zou, Jinling Meng, M Naveed Aslam, Jacqueline Batley, Wallace A Cowling, Derek Lydiate

**Affiliations:** 1 EH Graham Centre for Agricultural Innovation (an alliance between NSWDPI and Charles Sturt University), Wagga Wagga Agricultural Institute, Wagga Wagga, NSW 2650, Australia; 2 Diversity Arrays Technology Pty Ltd, 1 Wilf Crane Crescent, Yarralumla, Canberra, ACT 2600, Australia; 3 National Key Laboratory of Crop Genetic Improvement, Huazhong Agricultural University, Wuhan 430070, China; 4 School of Agriculture and Food Sciences, University of Queensland, St Lucia, QLD, Australia; 5 Agriculture and Agri-Food Canada, 107 Science Place, Saskatoon, SK S7N 0X2, Canada; 6 DNA Technologies Laboratory, National Research Council of Canada, 110 Gymnasium Place, Saskatoon, SK S7N 0X2, Canada; 7 School of Plant Biology, The University of Western Australia, 35 Stirling Highway, Crawley, WA 6009, Australia; 8 The UWA Institute of Agriculture, The University of Western Australia, 35 Stirling Highway, Crawley, WA 6009, Australia

## Abstract

**Background:**

Dense consensus genetic maps based on high-throughput genotyping platforms are valuable for making genetic gains in *Brassica napus* through quantitative trait locus identification, efficient predictive molecular breeding, and map-based gene cloning. This report describes the construction of the first *B. napus* consensus map consisting of a 1,359 anchored array based genotyping platform; Diversity Arrays Technology (DArT), and non-DArT markers from six populations originating from Australia, Canada, China and Europe. We aligned the *B. napus* DArT sequences with genomic scaffolds from *Brassica rapa* and *Brassica oleracea,* and identified DArT loci that showed linkage with qualitative and quantitative loci associated with agronomic traits.

**Results:**

The integrated consensus map covered a total of 1,987.2 cM and represented all 19 chromosomes of the A and C genomes, with an average map density of one marker per 1.46 cM, corresponding to approximately 0.88 Mbp of the haploid genome. Through *in silico* physical mapping 2,457 out of 3,072 (80%) DArT clones were assigned to the genomic scaffolds of *B. rapa* (A genome) and *B. oleracea* (C genome). These were used to orientate the genetic consensus map with the chromosomal sequences. The DArT markers showed linkage with previously identified non-DArT markers associated with qualitative and quantitative trait loci for plant architecture, phenological components, seed and oil quality attributes, boron efficiency, sucrose transport, male sterility, and race-specific resistance to blackleg disease.

**Conclusions:**

The DArT markers provide increased marker density across the *B. napus* genome. Most of the DArT markers represented on the current array were sequenced and aligned with the *B. rapa* and *B. oleracea* genomes, providing insight into the *Brassica* A and C genomes. This information can be utilised for comparative genomics and genomic evolution studies. In summary, this consensus map can be used to (i) integrate new generation markers such as SNP arrays and next generation sequencing data; (ii) anchor physical maps to facilitate assembly of *B. napus* genome sequences; and (iii) identify candidate genes underlying natural genetic variation for traits of interest.

## Background

Rapeseed (also known as canola and oilseed rape, *Brassica napus* L., 2n = 4× = 38; genomes AACC) is the second largest oilseed crop after soybean and provides a valuable rotational crop for farmers in many parts of the world. During the past 15 years, global oilseed rape production has doubled to >60 million tonnes (http://faostat.fao.org/; data sourced April 2012), due to the high demand for healthy vegetable oil, feedstock and a renewable source for the biodiesel industry. This amphidiploid *Brassica* species most likely originated as a result of repeated natural hybridization and genome doubling between the monogenomic diploid species *Brassica rapa* (2n = 2× = 20, genome AA) and *Brassica oleracea* (2n = 2× = 18, genome CC) along the Mediterranean coastline in Southern Europe, and was probably selected as an oilseed crop only 300–400 years ago [1-3].

Selection of useful variation in crop plants has been a major thrust of early farmers since the dawn of agriculture. In modern *Brassica* breeding programs, classical genetic analysis and molecular genetic approaches have been used to improve our understanding of the inheritance of various qualitative and quantitative loci and causative genes, to estimate their number, position and genetic effects, and to identify DNA-based markers associated with traits of agronomic importance [4].

Several linkage maps have been constructed from recombination data in *B. napus* mapping populations, which depict the distances between loci, as well as their order on a chromosome. These linkage maps have been based on a range of marker systems such as restriction fragment length polymorphisms (RFLPs), randomly amplified polymorphic DNAs (RAPD), amplified fragment length polymorphisms (AFLPs), simple sequence repeats (SSRs), sequence- tagged sites (STSs), sequence-related amplified polymorphisms (SRAPs) and single nucleotide polymorphisms (SNPs) [5-14]. These maps have been used to uncover qualitative and quantitative trait loci (QTLs) underlying traits of agricultural importance, for linkage disequilibrium assessment, candidate gene identification, marker-assisted selection, and genetic evolutionary studies [14-19]. The number of marker loci in these genetic linkage maps varied from 219 to 13,551, where the marker density was dependent upon the level of polymorphism between the parental lines of mapping populations and type of marker system used for detecting polymorphisms.

In order to increase marker density for predictive molecular breeding and map-based cloning of genes, dense consensus genetic maps have been developed for various crops including *B. napus*[14,20,21]. Such consensus maps are developed by mapping several common markers from each linkage group, in different mapping populations, and anchoring the linkage maps with consensus markers. However, most *B. napus* genetic maps have been based either upon low-throughput marker assays or on different marker systems, rendering them difficult to utilise to compare the map locations of markers and therefore their linkages with traits of interest across populations.

Diversity Array Technology (DArT) markers do not require prior DNA sequence information and can simultaneously genotype a large number of SNPs and insertion/deletion polymorphisms across the genome in a single assay, providing a low-cost option for genotyping [22]. The DArT marker system was first used in rice, and has since been extensively utilised for genetic applications such as genetic diversity analysis, construction of genetic linkage maps, and QTL/linkage analysis in different crops [23,24]. Recently, a 3072-clone *B. napus* DArT array was used to generate a linkage map using a *B. napus* doubled haploid (DH) population derived from Lynx-/Monty-028DH [13]. This information was used to identify QTLs for flowering time, resistance to blackleg disease caused by *Leptosphaeria maculans*, and resistance to pod shatter [25-27].

Comparative mapping studies have shown extensive co-linearity between the genomes of *Brassica* and *Arabidopsis*, which diverged from a common ancestor approximately 20 MYA [28-30]. The genome of *B. rapa* has recently been sequenced [31], whilst the genomes of *B. oleracea* and *B. napus* are currently being annotated and analysed and are expected to become publicly available in 2013. These advances in structural and functional genomics have provided us with an opportunity to align the genetic DArT markers with the sequenced genomes of *B. rapa* and *B. oleracea*. This allows the identification of markers for marker-assisted selection in rapeseed improvement programs.

In the present study, we constructed a consensus map of rapeseed based on DArT marker datasets produced using six DH *B. napus* populations. These populations were derived from Ag-Castle/Topas (AT), BLN2762/Surpass400 (BS), Lynx-037DH/Monty-028DH (LM), Maxol*1/Westar-10 (MW), Skipton/Ag-Spectrum (SAS), and Tapidor/Ningyou7 (TN). The parents of the mapping populations originated from Australia, Canada, China and Europe, and are currently being used internationally in rapeseed germplasm enhancement programs. We physically mapped the *B. napus* DArT sequences using genome sequences from scaffolds of *B. rapa* and *B. oleracea*. DArT marker loci associated with agronomic traits, including various components of phenology, plant architecture, seed and oil quality, nutrient uptake and mobilisation, and resistance to blackleg disease and abiotic stresses were also identified.

## Results

### Map construction from individual mapping populations

Individual component maps were constructed from six populations that were genotyped with DArT, along with a selection of SSR markers (Table 1, Additional file 1). The number of DArT marker loci on each map ranged from 217 (AT population) to 437 (LM population). The TN map consisted of the most markers (971); 403 DArT and 568 non-DArT (381 SSRs, 69 STSs including candidate genes, 68 SNPs, 34 RFLPs, 14 centromeric sequence-related and two AFLP markers). The genetic linkage map length for the six populations varied from 759.9 to 2,288 cM, with a mean length of 1446.5 cM.

**Table 1:** The distribution and density of markers in the linkage maps of individual doubled haploid populations

**Population**	**Total markers (no.)**	**DArT markers (no.)**	**Non-DArT markers (no.)**	**Length of map (cM)**	**Average marker interval (cM)**	Redundant markers* (%) DArT	**Non-DArT**
AT	217	217	0	759.9	3.50	47.5	NA
BS	363	295	58	1462.7	4.03	29.8	0
LM	586	437	149	2288.0	3.90	43.7	11.9
MW	285	285	0	1200.0	4.21	26.7	NA
SAS	275	275	0	1129.8	4.10	38.9	NA
TN	971	403	568	1838.4	1.89	9.2	0.7

* NA: not applicable.

The order of the DArT marker loci was generally similar among the different maps (Additional file 2). However, there was some evidence of chromosomal segmental rearrangements, such as inversions or translocations, on some genomic regions on chromosomes A7, A9, A10, C1, C2, and C6. For example, *XbrPb-658333* and *XbrPb-659113* were mapped within 0.5 cM on chromosome A10 in the TN population; however, these markers were not linked in the SAS population and were located on different linkage groups (A10-II and A10-III on the SAS map). In the LM population, a high frequency of homoeologous recombination was observed between chromosomes A7 and C6 [13]. The map lengths and marker densities were positively correlated among the populations (r = 0.73).

Segregation distortion was much more pronounced on chromosome A7 in the populations derived from AT (original data not shown), MW and SAS [32,33], where either targeted selection for desirable alleles (for example for different blackleg resistance genes *Rlm1*, *Rlm3* and *Rlm4*) might have occurred within the breeding programs or could have been a low level of homoeologous interaction with chromosome C6.

The DArT markers were generally well distributed throughout the genome, although some chromosomes exhibited either no or low polymorphism for the DArT markers (Additional file 2). For example, chromosome A3 had low DArT marker density (4 DArT/35 total markers) in the TN map. A number of DArT markers co-segregated and therefore mapped on as the same locus. DArT marker redundancy (markers that map to the identical map position as their respective neighbouring markers) ranged from 9.2% in the TN to 47.5% in the AT mapping population (Table 1). In order to estimate redundancy of the DArT markers at the sequence level, we aligned the DArT sequences using ClustalW program and found that the majority of co-segregating DArT clones (0.01 cM = ~6 kb) had high levels (77% to 99.4%) of sequence identities (Additional file 3). However, a marker cluster on chromosome C3 had lower sequence identities (53 to 80%) and hence may not represent the same locus. Co-segregation of markers may have occurred because recombination is difficult to observe between loci that are very close to each other, particularly in the small populations used here (Table 2).

**Table 2:** Attributes of doubled haploid genetic mapping populations used for consensus map construction

**Parentage**	Source*	**Code**	**size**	**Markers**	**Segregating Traits**	**Reference**
Ag-Castle/Topas	AAFC, Canada	AT	71	DArT	Resistance to *L. maculans*	Larkan et al. (unpublished)
BLN2762/Surpass400	NSWDPI	BS	134	DArT, Non-DArT based	Resistance to *L. maculans*	Raman et al. [26]
					Resistance to shatter	Raman et al. (unpublished)
					Water soluble carbohydrates	Raman et al. (unpublished)
Lynx-037DH/Monty-028DH,	UWA	LM	131	DArT, Non-DArT	Flowering time, seed oil quality	13, 43, This study
Maxol*1/Westar	VICDPI	MW	100	DArT	Resistance to *L. maculans*	26, 33
Skipton/Ag-Spectrum	NSWDPI	SAS	172	DArT, Non-DArT based	Various component of flowering time	27
					Race-specific and non-specific resistance to *L. maculans*	32
					Carbon isotope discrimination	Luckett et al. (unpublished)
					Water soluble carbohydrates	Raman et al. (unpublished)
Tapidor/Ningyou7	HAU	TN	153	DArT, Non-DArT based	Various component of flowering time	15, 34, 37, 40
					Oil content	9
					Erucic acid	9
					α-tocopherol content	41
					Glucosinolate concentration	9, 35

*AAFC: Agriculture and Agri-Food Canada (Saskatoon), HAU: Huazhong Agricultural University, Wuhan, China, NSWDPI: New South Wales Department of Primary Industries, UWA: University of Western Australia, VICDPI: Victorian Department of Primary Industries.

### Consensus map construction

Individual component maps were aligned to the reference linkage map of the TN-DH population to construct a consensus map. A total of 1359 marker loci [791 DArTs, 381 SSRs, 69 STSs, 68 SNPs, 34 RFLPs, 14 centromeric region specific (CS) and two AFLPs] were integrated in the consensus map, covering 1987.2 cM, with an average interval distance of 1.46 cM, across the 19 chromosomes of *B. napus* (Table 3, Additional file 4). The A and C genomes had genetic lengths of 1062.5 cM and 924.6 cM, with the mean distances between neighbouring markers of 1.08 cM and 2.48 cM, respectively. Approximately 2.6 times more markers were mapped on the A genome (986) than the C genome (373), suggesting that the A genome was more polymorphic than the C genome in *B. napus.*

**Table 3:** The distribution and density of markers across 19 rapeseed chromosomes of the consensus map

**Chromosome (Linkage group)**	**Number of marker in consensus map**	**Map length (cM)**	**Mean distance between markers (cM)**	**No of marker-gaps >10 cM**	^*^Redundancy in DArT marker (%)	**Redundancy in Non-DArT markers (%)**
A1	110	123.6	1.12	2	19/71 (26.8%)	1/39 (2.6%)
A2	70	90.5	1.29	1	13/48 (27.1%)	0/22 (0%)
A3	156	144.9	0.93	-	22/85 (25.9%)	1/71 (1.4%)
A4	97	102.3	1.06	-	12/81 (14.8%)	0/16 (0%)
A5	92	82.0	0.89	-	14/75 (18.7%)	0/17 (0%)
A6	118	107.7	0.91	-	21/91 (23.1%)	1/27 (3.7%)
A7	81	115.3	1.42	1	7/34 (20.6%)	1/47 (2.1%)
A8	35	66.9	1.91	-	1/17 (5.9%)	0/18 (0%)
A9	145	128.3	0.89	-	23/81 (28.4%)	0/64 (0%)
A10	82	101.1	1.23	1	11/54 (20.4%)	0/28 (0%)
A genome (total)	986	1062.5	1.08	5	143/637 (22.4%)	4/349 (1.1%)
C1	42	63.8	1.52	-	9/22 (40.9%)	0/20 (0%)
C2	51	110.2	2.16	2	8/35 (22.9%)	0/16 (0%)
C3	76	153.9	2.03	1	9/30 (30.0%)	1/46 (2.2%)
C4	45	132.9	2.95	1	2/21 (9.52%)	0/24 (0%)
C5	24	93.9	3.91	1	1/7 (14.3%)	0/17 (0%)
C6	42	98.8	2.35	3	0/11 (0%)	0/31 (0%)
C7	23	59.5	2.58	-	0/8 (0%)	0/15 (0%)
C8	31	103	3.32	2	0/8 (0%)	0/23 (0%)
C9	39	108.6	2.79	1	2/12 (16.7%)	0/27 (0%)
C genome (total)	373	924.6	2.48	11	31/154 (20.1%)	1/219 (0.5%)
AC genomes (total)	1359	1987.2	1.46	16	174/791 (22.0%)	5/568 (0.9%)

^*^Redundancy refers to co-location of markers at the same genetic locus.

The length of individual chromosomes varied from 59.5 cM (chromosome C7) to 153.9 cM (chromosome C3), whereas the number of marker loci per chromosome ranged from 23 (chromosome C7) to 156 (chromosome A3). Chromosomes A5 and A9 had the highest marker density (1 marker/0.89 cM), and chromosome C5 had the lowest marker density (1 marker/3.91 cM). Both map length and marker density across chromosomes were moderately correlated (r^2^ = 0.56, Additional file 5).

In general, the consensus map had good coverage of DArT and non-DArT markers, however there were 16 genomic sites (accounting for 6% of the genetic map) where adjacent markers were >10 cM apart: on chromosomes A1, A2, A7, A10, C2, C4, C5, C6, C8 and C9 (Table 3, Additional file 4). Of the 791 genome-wide DArT markers, 22% showed redundancy, whereas only 0.9% of non-DArT markers showed redundancy*.* Chromosomes A6 and C1 exhibited the maximum redundancy of 3.7% and 40.9% for non-DArT and DArT markers, respectively (Table 3). The overall marker density on the consensus map (1 marker/1.46 cM) was higher compared to that observed in individual populations (1 marker/1.89 - 4.20 cM) (Tables 1 and 3). A total of 10 major clusters of DArT markers (≥5 loci/0.01 cM) were identified on chromosomes A1, A2, A3, A6, A9, and C1 (Additional file 4).

### Comparison of individual maps and the consensus map

The congruency of marker order and map positions was consistent with an earlier map of TN [9]. The positions of the DArT markers on the consensus map were generally consistent with their locations on the six individual linkage maps from the biparental crosses (Additional file 2). The order of markers was moderately correlated in most pair-wise comparisons of populations, and highly correlated between the AT and SAS populations (Additional file 6). However, there was a poor correlation between the BS and LM populations (r < 0.4). Due to the diverse backgrounds of the DH populations used in this study, the number of shared DArT markers was extremely variable and ranged from 0 to 27 per chromosome. The largest number of shared markers per chromosome (27) was between the LM and BS maps of chromosome A3, covering a genetic distance of ~123 cM. Spearman’s rank correlation of the marker orders also varied between different component maps (Additional file 6). Therefore, it was difficult to reliably compare the marker orders across individual maps.

### Colinearity of the consensus map and the sequence assemblies of A/C ancestral species

The genomic locations of DArT markers and their homology (sequence identities and bit scores) are provided in Additional files 7 and 8. Approximately 80% (2,457/3,072) of DArT markers were physically mapped to the sequenced genomes of *B. rapa* and *B. oleracea*. This enabled evaluation of the correspondence of the DArT markers on the genetic and physical maps. The location of 615 DArT clones on the genome scaffolds of *B. rapa* and *B. oleracea* (256 on the A genome and 359 on the C genome) could not be determined, as they did not return any hit. These sequences may represent unassembled regions of the current genome assemblies or alternatively may be divergent *B. napus* sequences (Additional file 9). The alignment of the integrated genetic and physical maps was generally in agreement (Additional file 10); however, in a number of instances marker order as determined by sequence homology suggested chromosomal rearrangements on the linkage groups A3, A9, C2 and C4 and or due to inaccuracies in mapping. For two linkage groups, C7 and C8, the alignment of the DArT markers to the genome scaffolds suggested that significant portions of these two chromosomes were not polymorphic in the six crosses.

### Distribution of marker duplications across A and C genomes

Eighty-three markers (18 DArT and 65 non-DArT) showed inter- and intra-genomic duplicated loci on the homoeologous chromosomes of the A and C genomes (Additional files 4 and 11). The frequency of duplicated (multi-locus) markers varied from 2.27% (18/791) to 11.4% (65/568) for DArT and non-DArT markers, respectively, in the consensus map. Chromosome A9 had the most duplicated marker loci (13), followed by A2 (10), whereas C1 and C5 did not contain any duplicated marker loci. In addition, up to five alleles for the marker *Xpw123* could be mapped on chromosomes A6, A9 and C8. Several DArT markers also showed multiple sequence identities with the sequences of *B. rapa* and *B. oleracea* localised on different chromosomes (Additional files 7 and 8).

### Association between DArT markers and agricultural traits

We surveyed all genetically mapped markers, including DArT, that showed association with traits of agricultural importance in the mapping populations. This included traits from the published literature, along with fatty acid quality QTLs, published for the first time here. In total 139 marker intervals were identified, associated with resistance to Sclerotinia and *L. maculans* (causing blackleg disease), yield related traits (seed number, seed weight, seed yield, oil yield, branch number, and pod number), oil quality traits (glucosinolate content, fatty acid content, and vitamin E), biomass yield, phenology traits (plant height, flowering time, vernalisation requirement, maturity time), male sterility, boron efficiency, and uptake of nutrients, including boron, calcium, iron, magnesium, copper, zinc, and phosphorus (Additional files 4 and 11) [9,27,32-47]. We did not attempt to locate all (246) QTLs that were reported to be involved in the glucosinolate metabolic pathway in the TN population [35]. Details of four newly identified QTLs governing 16:0, 18:1, 18:2 and 18:3 fatty acids along with five correlated QTLs for 20:1, 20:2, 22:0, 22:1 and 24:0 in the LM population are presented in Table 4. These highly significant QTLs accounted for 13.9% to 75.6% of the explained variation. The marker interval *XbrPb*-*X657955* - dFAD2a explained the highest (75.6%) variation, whilst the marker interval brPb-*662948* - *brPb-660893* (13.9%) explained the lowest amount of variation in linolenic acid and palmitic acid content, respectively. The fatty acid desaturase-2 (*FAD2*) gene based marker was mapped within two of the QTLs on chromosome A5. A majority of the qualitative and quantitative trait related loci identified in this study, resided within 10 cM of DArT marker intervals. Some previously published QTLs could not be precisely identified due to the lack of common shared markers between the consensus map and the original mapping population and hence they were defined as approximate (Additional file 12).

**Table 4:** Newly identified QTL for fatty acid seed content in the LM population

**Fatty acid**	**Chromosome**	**Location in LM map (cM)**	SD*	**Marker interval**	**LOD**^**#**^	SD*	**PEV**^**$**^	SD*	**Other correlated QTL**
16:0 (Palmitic acid)	A01	62.6	12.6	XbrPb-662948 - XbrPb-660893	3.71	1.48	13.9%	0.051	
18:1 (Oleic acid)	A05	94.9	3.1	XbrPb-657955 - XdFAD2a	20.14	4.31	64.0%	0.083	20:1, 20:2
18:2 (Linoleic acid)	A05	94.2	1.2	XbrPb-657955 - XdFAD2a	30.78	3.86	75.6%	0.035	
18:3 (Linolenic acid)	C4	141.6	1.4	XsN11516 - XsN0704	15.79	2.86	45.8%	0.057	22:0, 22:1, 24:0

* SD = Standard deviation; ^#^LOD = logarithm of the odds; ^$^PEV = Proportion of explained variation.

## Discussion

In this study, we developed the first consensus genetic map of the rapeseed genome based on DArT markers. The development of this consensus map will provide a platform to compare chromosomal locations of markers across rapeseed populations and facilitate identification of simple and complex inherited trait-marker associations, comprehensive assessment of genetic diversity, and whole genome selection in rapeseed breeding programs. The consensus map consisted of 1,359 markers spanning all 19 *B. napus* chromosomes, with an average marker density of one marker per 1.46 cM. This corresponds to approximately 880 Mbp of the *B. napus* genome, 94% of the mapped AC genome was contained within intervals <10 cM. These observations suggest that this consensus map will be suitable for various applications including detection of quantitative traits in rapeseed improvement programs, as in most QTL analyses a 10-cM interval between marker loci is commonly used for regression analysis. This consensus map based on the sequenced DArT markers will also allow the positional cloning of the causative genes controlling phenotypic variation, estimation of linkage disequilibrium at the individual chromosome/genome level, and identification of genomic rearrangements such as translocations that occurred during the period between the genome triplication of *Brassica* and the divergence of *B. rapa* and *B. oleracea* and their distant relative model plant, *Arabidopsis*.

Different marker systems were used in each population, for example, the LM population was mapped with DArT markers, it had the highest density of SSR markers. These SSR markers (developed by the Agiculture and Agri-Food Canada Consortium) were not used in any other population undertaken in this study except for the TN population. The TN population was also mapped with a variety of markers based on SNPs and CS, which were not used in any other population. Furthermore, DArT and SSR markers amplify intra- and inter-homoalleles as a result of chromosomal duplications between homoeologous sequences among subgenomes and between paralogous duplicated sequences and other rearrangements such as in the TN (Additional files 4 and 11) and SAS populations [35,48], which makes it difficult to compare individual component maps. Of the DArT clones that were useful for mapping across the six segregating populations in this study, relatively few loci were common between populations. This could be due to utilisation of a range of DH populations derived from genetically diverse parental lines used in order to map the DArT clones across the *B. napus* genome. For example, the cultivar Surpass 400 is derived from several backcrosses into cultivated *B*. *napus* from *B. rapa* ssp. *sylvestris*. The introgression of chromosomal segments from *B. rapa* into Ningyou7 – one of the parental lines of the TNDH population - has also been recently reported [49]. We could not ascertain the map positions of some DArT loci or their order on certain chromosomes (for example in the MW population, LG I to VI, Additional file 1) due to the absence of shared markers among mapping populations. However, marker order was generally consistent within some chromosomes where more than four shared markers were present for assessment of statistical significance [21] and there was a global conservation of co-linearity between the corresponding linkage groups of integrated maps and the A (*B. rapa*) and C (*B. oleracea*) genome scaffolds. On some chromosomes no polymorphism was found between the parental lines of the DH populations, for example on chromosome C8 in the populations MW and BS. These findings suggest that the DArT sequences in the genomic representations were conserved.

The results presented here demonstrate that the consensus map was longer (1,987.2 cM) than the six individual maps with the exception of the LM population. The LM map contained a higher proportion of distorted and distinct loci [13], than most of the populations. The recombination frequency can also vary with genetic background [48]. In addition, scoring errors tend to be higher with SSR markers which show extensive stuttering and multiple bands (alleles), as compared to DArT markers. These biological and non-biological factors could have skewed the map estimates.

In general, the DArT markers were well-distributed across the genome, however, certain chromosomal regions showed extensive clustering such as on chromosomes A9 and C2 (Additional file 4). This may indicate the presence of gene-rich regions and uneven distribution of recombination events along chromosomes, or it may suggest that the DArT markers are preferentially surveying DNA polymorphisms that are unevenly distributed along chromosomes. Similar results have been reported in other crops including in *B. napus*[14,49,50]. Uneven distribution of DArT markers between the A and C genomes may indicate genome evolution events of *B. napus* from its diploid progenitor species or uneven representation of DArT clones from source species used in the array development. The DArT markers derived from genomic representations are known to introduce some degree of redundancy [22]. However, in this study, genomic representations were prepared using the CNG methylation sensitive restriction enzyme *Pst*I, which generates low and single copy DNA fractions in plants.

In comparison to the non-DArT markers (11.4%), a low proportion (2.27%) of multi-locus DArT markers was found. Hybridisation-based markers (such as DArT) select against multi-locus markers, because hybridisation intensities contributed by different loci are difficult to resolve in the DArT allele calling process and such markers are normally scored as monomorphic. A similar frequency (~1.8%) of multi-locus DArT markers has been reported in rye, sorghum, and barley [22,50,51]. Existence of multi-locus markers as a result of DNA (DArT) hybridisation from orthologous and non-homologous regions of rapeseed, and multiple hits between DArT sequences and the sequenced scaffolds of *B. rapa* and *B. oleracea*, provide strong evidence for the presence of intra- and inter-chromosomal duplicated loci in *B. napus*. Previous studies have reported that most of the approximately 1.2 Gb genome of *B. napus*[52] comprises genome sequences from the two progenitor species, which exhibit significant co-linearity. However, homoeologous recombination plays a major role in chromosome rearrangements, such as duplications and reciprocal translocations [53-57]. In certain cases, the physical position of DArT markers on the *B. rapa* and *B. oleracea* scaffolds did not correspond with their genetic positions on the linkage maps of segregating populations as reported previously [58]. This could have been caused by large translocations within the mapping populations, mapping inaccuracies and/or errors in assembling genome scaffolds. However, there was a good correspondence between genetic ordering and the current genome sequence assemblies. Approximately 20% of the DArT marker sequences did not have a significant match to *B. rapa* and *B. oleracea*. This may be due to the incomplete genome coverage in the current genomic scaffolds or some of them may represent novel loci that may have evolved in *B. napus* as a result of inter-specific recombination between *B. rapa* and *B. oleracea.* However, a more likely explanation is biological (structural) variation among ancestral genotypes involved in the development of *B. napus* cultivars used for mapping, as compared to *B. rapa* and *B. oleracea* genotypes used for sequencing the A and C genomes [58]. It is also possible that the reference accessions used to build current A and C genome scaffold assemblies lack some genomic regions that are present in the wider gene-pools.

Discrepancies such as inversions and translocations were identified when comparing the consensus map and the individual component maps, as have been reported previously in *B. napus*[59] and in comparisons of the A genome in *B. napus* and *B. rapa*[58]. Apart from genome rearrangements, this could be due to the smaller size of the populations used here [60], and variation in the number of recombination events in different regions of the plant genomes [61].

A recent study has shown good co-linearity of DArT and non-DArT markers between the genetic linkage map of the SAS population and the assembled pseudomolecules of *B. rapa*[27]. As the sequenced genome scaffolds of other A and C genome *Brassica* species become available in the public domain, DArT sequences (available on http://www.diversityarrays.com/) can be used for comparative genomic analysis, identification of QTLs for predictive breeding (as shown in this study) and for the identification of candidate genes linked to traits of agricultural importance.

In this study, we related DArT markers with the majority of QTLs that have been identified in six breeding populations, however this can be extended to other traits and populations of *B. napus,* as well as to related A and C genome *Brassica* species. For example, several DArT markers were related to the positions of QTLs associated with flowering and quality components in the TN and SAS populations [9,15,27,37,40]. Two QTLs for flowering time were mapped in the genomic regions harbouring homoeologues of *FLOWERING TIME LOCUS C* of *Arabidopsis, BnFLC.A3* on chromosomes A3 and *Bn.FLC.A10* on chromosome A10 [15,27,40]. In the LM population, we identified four QTLs associated with 16:0, 18:1, 18:2 and 18:3 fatty acids on chromosomes A1, A5 and C4 (Table 4). Among them, two major QTLs associated with 18:1 (oleic acid) and 18:2 (linoleic acid) were mapped on A5 within a region containing *FAD2* gene (Table 4). Localisation of these QTLs suggested that *FAD2* controls variation for both oleic acid and linoleic acid content in this population. The *FAD2* of the endoplasmic reticulum encodes ω-6 desaturases which is responsible for conversion of oleic acid to linoleic acid by inserting a double bond at the ω-6 position [62].

The parents of mapping populations used in this study such as Ag-Castle, Maxol, Monty, Skipton, Ag-Spectrum, and Surpass 400 are known to harbour resistance both at the seedling (race-specific, qualitative) and adult plant stages (race-specific and/or race-non-specific) to *L. maculans*[26,32,63]. DArT markers were mapped in the vicinity of a genomic region associated with *Rlm1, Rlm3,* and *Rlm4* genes for resistance to *L. maculans* on the chromosome A7 [32,33]. It was interesting to note that some of the loci associated with correlated traits such as seed yield, pod number, seed weight, flowering time, seed number and plant height were localised on the same genomic regions (A1, A2, A3, A7, C3 and C6) on the consensus map, which may have been due to pleiotropic effects of a QTL on different traits and/or tight linkage between multiple traits and QTLs [64].

## Conclusions

We constructed a high-density consensus map of *B. napus* utilising 1,359 DArT and non-DArT based markers. This consensus map was useful in locating loci associated with various traits of agronomic importance. The sequences of the DArT clones are publicly available (http://www.diversityarrays.com/) and provide a valuable resource in predicted genomic breeding, map-based gene cloning, and comparative analysis studies of A and C genomes *Brassica* species. The development of the integrated consensus map described in conjunction with the physical locations of *B. napus* DArT markers on the genome scaffolds, and the identification of molecular markers flanking genomic regions associated with agronomic traits, will empower rapeseed breeding programs to identify candidate/causative genes controlling genetic variation for such traits. This will enhance selection efficiency, especially of quantitative traits governed by a large number of genes and influenced with G × E interaction via marker-assisted selection.

## Methods

### Mapping populations

Six DH populations derived from 12 parents were used for consensus map construction. The structure of these populations and the traits that were studied are summarised in Table 2. We used the TN-DH population from parents Tapidor (Winter-type, European cultivar) and Ningyou7 (semi-winter, Chinese cultivar) as a reference, because this population has been extensively used by the international rapeseed community for the genetic/comparative mapping of a range of traits of agronomic importance such as plant architecture, flowering time, seed and oil quality attributes, boron efficiency, and glucosinolate content [9,15,34-38]. The TN-DH population has been used to map 614 non-DArT markers based upon SSRs, SNP, STSs, single strand conformation polymorphisms (SSCPs), RFLPs, cleaved amplified polymorphic sequences (CAPSs), and AFLPs, as described previously [9,35,39] and with SNPs identified from the rapeseed transcriptome [49]. This population has resulted in the discovery and mapping of candidate genes for sucrose transporter, α-tocopherol, fatty acid elongase, indehiscent (*IND*) gene for pod shatter, and *FLOWERING LOCUS C (FLC)*, *FLOWERING LOCUS T*, *APETALA2* for flowering time [15,34,40-42,65]. The TN-DH published map was further saturated with DArT markers in this study.

We used the published map data of Lynx-037DH/Monty-028DH (LM) population [13]. The LM map was constructed utilising DArTs, SSRs, intron polymorphism (IP), and candidate gene-based markers for fatty acid desaturase and *FLC* genes. Other populations derived from AT, BS, MW, and SAS were genotyped predominantly with DArT markers and their individual component maps were developed in this study.

### Trait phenotyping

Seed fatty acid quality (specifically 14:0, 16:0, 16:1, 18:0, 18:1, 18:2, 18:3, 20:0, 20:1, 20:2, 22:0, 22:1, 24:0 and 24:1 fatty acids) was measured in the LM DH population using gas chromatography (GC; Perkin-Elmer Auto System Gas Chromatograph; Waltham, MA, USA) using 1.5 g of seed per line as described previously [43]. DH lines were grown as spaced plants in two partially replicated spaced plant nurseries at Manjimup Horticultural Research Centre (Manjimup, WA, Australia) in the summer or 2004/05 and at Shenton Park Field Station (Perth, WA, Australia) in the winter of 2005, with 12 DH lines included in both nurseries. Self-pollination was enforced by enclosing racemes in perforated plastic bread bags.

### DNA isolation

DNA was isolated from the fresh leaf samples of 2-3-week-old seedlings from parental lines and their progenies using a standard phenol/chloroform extraction method [66].

### DArT marker analyses

All six populations were genotyped with the *B. napus* version 1.0 DArT microarray containing 3,072 markers that were selected after genotyping several hundreds of *B. napus* accessions at Diversity Arrays Technology Pty Ltd (DArT P/L, Yarralumla, ACT, Australia) as described previously [13]. The *Pst*I*/Bst*NI genomic representations of individual samples (parental lines and their segregating derivatives) were generated, labelled with fluorescent dyes (Cy3 and Cy5) by random priming [67], and hybridised with the *B. napus* DArT array. Images of microarrays were acquired using a scanner (Tecan LS300; Grodig, Salzburg, Austria) and further analysed with the DArTsoft software version 7.4.7 (DArT P/L). The same software was used to score polymorphisms among parental lines and their DH lines in a binary format (for the presence of marker in the representation as ‘1’ and for the absence as ‘0’, as described previously [13,22]. For quality control, two measures were used: the first was based on the Q value (a quality parameter measuring bimodality of signal distribution between ‘0’ and ‘1’ clusters) and the second was based on call rate (P, the percentage of DNA samples with defined ‘0’ or ‘1’ allele calls). Only high quality DArT clones with Q > 77%, a call rate >97% and 100% allele-calling consistency (reproducibility) across the technical replicates were selected as markers for genetic mapping of different DH populations. However, some lower quality (those with less well supported map positions) were integrated as attached markers into the most appropriate positions in framework component maps, as described previously in the LM population [13].

### PCR-based marker analysis

Both publicly and privately available SSR primer-pairs with prefixes ‘BRMS’, ‘BRAS’, ‘CB’, ‘MR’, and ‘MD’ were obtained from the literature [5,68,69]. Two assays; capillary electrophoresis and agarose gel electrophoresis were performed for allele sizing of SSR amplicons generated with fluorescently labelled and non-fluorescent primers, respectively. The 5’ ends of the forward primers were tailed with 19-bp long M13 sequences and labelled with a fluorescent dye (D2, D3 or D4; Beckman Coulter Inc., Fullerton, USA) as previously described [64,70]. SSR primers were synthesised by Sigma-Aldrich (Castle Hill, NSW, Australia) and were diluted to 1 to 10 pmol/μl depending upon the assay used. PCRs and amplifications were carried out as described previously [64]. Amplified DNA fragments (>600 bp) were separated on a CEQ8000 DNA sequencer (Beckman Coulter Inc., Brea, CA, USA) and their sizes measured using fragment analysis software. Fragments were scored by allele sizes in bp and then converted into binary format as described above. We adopted the standard nomenclature for the DArT markers as described previously [13].

### Linkage map construction

Individual genetic (component) maps of the six populations were constructed separately using DArT linkage group and marker ordering software [71]. Prior to construction of the map, the redundant markers were ‘binned’, and within each bin the marker with the highest quality was used for map construction.

The DArT marker ordering system followed a three-step process similar to the one described previously [72]. In the first step, the ‘first’ markers were grouped. A complete graph of all the markers was constructed where the weight assigned to the edges corresponded to the recombination frequency. Edges corresponding to a recombination frequency above a threshold (typically around 0.2) were removed and the resultant connected components were collected into linkage groups. In the second step, the markers in each group were ordered. The optimum marker order was posed as the travelling salesman path within the group [73]. The optimal solution was based on the Concorde solver. Finally, the marker positions were converted to centiMorgans (cM) by applying the Kosambi function [74] to the recombination frequencies.

The reference map of TN was compared with the map constructed using software JoinMap 4.0 [75] with a threshold LOD score of 3.0, however the minimum LOD score of 1.4 was used to incorporate some markers. Non-DArT based markers (SSRs, SNPs, STSs, SSCPs, RFLPs, CAPSs, and AFLPs) with known chromosomal locations on the TN genetic linkage map were used to assign linkage groups to chromosomes A1-A10 and C1-C9 representing AA and CC genomes, respectively. Subsequently, this reference map was used to assign the chromosomal positions for DArT markers in other described mapping populations.

Allele segregation ratios for each marker locus were determined using χ2 tests to determine whether they conformed to expected Mendelian ratios for one locus (1:1 allelic ratio) or two locus (1:3 or 3:1 allelic ratio) models [13]. Markers that showed significant segregation distortion (P < 0.01) were discarded and were not used for linkage mapping.

### Consensus map construction

The segregation marker data and their order determined for each population were integrated into the consensus map with the DArT consensus map software [71]. The software required the following inputs: (i) seed (reference) map with one linkage group and marker positions per chromosome; and (ii) set of linkage groups from individual populations with marker positions and chromosome assignment for each group. The construction procedure used the following pseudo-code algorithm: (i) initialised consensus map with the seed map; and (ii) for each chromosome, we found a subset of linkage groups for this chromosome and repeated until subset was empty for each group. The process required at least three markers in common with the consensus map and correlated the positions of common markers with the consensus map. The group with the highest commonality [correlation × log (number of common markers)] was identified, and if the correlation was larger than 0.5, all markers were joined to the consensus map by linear interpolation. The group was removed from the subset and the process repeated. The individual and consensus maps were visualised graphically using the software MapChart [76].

### Sequence analysis of DArT clones

All 3,072 DArT markers that represented the current *B. napus* DArT array (version 1) were sequenced using ABI Prism Big Dye Terminator Cycle Sequencing Ready Reaction Kit (Perkin Elmer, MA, USA) and analysed on an ABI DNA Sequencer. The clones were sequenced using universal M13 forward primer. The sequences were analysed with the dedicated pipeline developed by Diversity Arrays Technology Pty Ltd (unpublished).

### *In silico* mapping of DArT markers on the *B. rapa* and *B. oleracea* genomes

Sequences of DArT clones were used to search the assembled *B. rapa*[31] and *B. oleracea* (Isobel Parkin and Andrew Sharpe, planned for publication) genome scaffolds using BLASTN [77] and the predicted marker positions were compared with their locations on the consensus genetic map. The vector and adapter sequences were trimmed from DNA sequences before determining the percentage sequence identities. We recorded the percentage identity using an E-value 10^-5^ threshold of all scoring pairs rather than recording only the best hit, in order to capture genome-wide sequence similarities.

### Detection of DArT sequence redundancy

In order to estimate redundancy at the sequence level, we aligned the DArT sequences with a multiple alignment algorithm CLUSTALW [78] implemented in Geneious software [79], with the default option for analysis of gaps. Although it is difficult to establish a threshold to declare a DArT marker redundant, we used a pragmatic approach and assumed that markers showing <80% sequence homology were less likely to be identical, because they should not hybridise to the same targets under stringent hybridisation conditions.

### Identifying trait-marker associations and chromosomal (marker) rearrangements

The consensus map developed in this study was used to (i) localise QTLs and major genes that were identified in the six DH populations; and (ii) identify chromosomal rearrangements such as duplications and inversions on the basis of markers. The location of genetic markers on the consensus map was delimited by identifying published common markers flanking the qualitative and quantitative loci in the individual genetic mapping population. QTL analysis of seed fatty acid content in the LM population was performed using MultiQTL v2.5 (MultiQTL Ltd, Haifa, Israel) using default parameters. The goodness of fit of one-gene models was compared to those of two-gene models.

## Competing interests

The authors declare that they have no competing interests.

## Authors’ contributions

HR and AK coordinated and designed the study. HR, RR, MNN, and FD, wrote the manuscript. RR, YL and MNA isolated DNA samples and generated the PCR marker data of mapping populations. AK, FD and HR analyzed the DArT data and constructed genetic and consensus maps. HR, JZ, YL and JM compiled published trait-marker associations. DE, IP, JB and AS performed *in silico* physical mapping with A and C genome scaffolds. HR investigated DArT sequence analysis. MNA, WAC and MNN analysed fatty acid profiles and conducted QTL mapping in the LM population. NL and DL provided AT mapping population. All the authors have commented, read and approved the final manuscript.

## Supplementary Material

Additional file 1 Molecular markers and their map positions on the genetic linkage map of the doubled haploid populations and consensus map. Click here for file

Additional file 2 Comparative linkage map of different chromosomes constructed from the individual six doubled haploid populations derived from Ag-Castle/Topas (AT), BLN2762/Surpass400 (BS), Maxol*1/Westar-10 (MW), Lynx-037DH/Monty-028DH (LM), Skipton/Ag-Spectrum (SAS) and Tapidor/Ningyou7 (TN), respectively (for enlarged view × 1.5). Click here for file

Additional file 3 Percent sequence identities of DArT clones that map in a cluster on different chromosomes of the consensus map. Click here for file

Additional file 4 Consensus map of *B. napus *constructed from the six doubled haploid populations derived from Ag-Castle/Topas, BLN2762/Surpass400, Lynx-037DH/Monty-028DH, Maxol*1/Westar-10, Skipton/Ag-Spectrum, and Tapidor/Ningyou7, respectively (for enlarged view × 5). Click here for file

Additional file 5 Comparisons between marker density and map length (in cM) in the consensus map developed from six component individual maps. Click here for file

Additional file 6 Shared markers across different mapping populations. Click here for file

Additional file 7 **Sequence identities of DArT clones with the genome scaffold of *Brassica rapa.*** Click here for file

Additional file 8 **Sequence identities of DArT clones with the genome scaffold of *Brassica oleracea.*** Click here for file

Additional file 9 **List of DArT clones that did not show any 'hit' with the genome scaffolds of *Brassica rapa *and *Brassica oleracea.*** Click here for file

Additional file 10 **Relationship between genetic map distance and physical map distance for all the 19 *Brassica* A and C genome chromosomes.** Genetic distances are given in cM, derived from the *B. napus* consensus map. Map order of linkage groups was orientated according to genome scaffolds. DArT sequences which were genetically mapped on to linkage groups were only aligned against the corresponding scaffolds. DArT sequences those showed multiple sequence alignments within the linkage group (chromosome) were also included. Click here for file

Additional file 11 List of inter-and intra homoalleles of marker loci mapped on the consensus map developed from six mapping populations. Click here for file

Additional file 12 Chromosomal position of molecular markers associated with various traits segregating in the mapping populations used for construction of consensus map. Click here for file
