# Supplementary material for: A consensus map of rapeseed (Brassica napus L.) based on diversity array technology markers: applications in genetic dissection of qualitative and quantitative traits
Source: BMC Genomics. 2013 Apr 23;14:277. doi: 10.1186/1471-2164-14-277 (PMC3641989; doi:10.1186/1471-2164-14-277)
Supplement: Additional file 2 — Comparative linkage map of different chromosomes constructed from the individual six doubled haploid populations derived from Ag-Castle/Topas (AT), BLN2762/Surpass400 (BS), Maxol*1/Westar-10 (MW), Lynx-037DH/Monty-028DH (LM), Skipton/Ag-Spectrum (SAS) and Tapidor/Ningyou7 (TN), respectively (for enlarged view × 1.5). [file 1471-2164-14-277-S2.rtf]

Additional file 2. Comparative linkage map of different chromosomes constructed from individual six doubled haploid populations. Homologous marker loci are highlighted in bold and connected with bold lines adjacently between linkage groups. Original map positions are given in Additional file 1.  
