# Supplementary material for: A consensus map of rapeseed (Brassica napus L.) based on diversity array technology markers: applications in genetic dissection of qualitative and quantitative traits
Source: BMC Genomics. 2013 Apr 23;14:277. doi: 10.1186/1471-2164-14-277 (PMC3641989; doi:10.1186/1471-2164-14-277)
Supplement: Additional file 3 — Percent sequence identities of DArT clones that map in a cluster on different chromosomes of the consensus map. [file 1471-2164-14-277-S3.rtf]

Additional file 3: Percent sequence identities of DArT clones that map in a cluster on different chromosomes of the consensus map of rapeseed.
Chromosome A2 cluster
Consensus map position (cM)	DArT
Locus	Sequence identity (%)	
		brPb-657614	brPb-657868	brPb-659016	brPb-659053	brPb-661407	brPb-662239	brPb-662312	brPb-670471	
28.62	brPb-657614	100	60.5	60.7	60.1	49.9	60.4	60.7	48.6	
24.96	brPb-657868	60.5	100	99.5	98.6	86.4	99.5	99.1	95.1	
24.95	brPb-659016	60.7	99.5	100	99.1	87	100	99.2	96.8	
24.96	brPb-659053	60.1	98.6	99.1	100	87.6	98.9	97.7	95.9	
24.96	brPb-661407	49.9	86.4	87.0	87.6	100	85.0	82.4	98.4	
24.96	brPb-662239	60.4	99.5	100	98.9	85.0	100	97.8	87.2	
24.95	brPb-662312	60.7	99.1	99.2	97.7	82.4	97.8	100	85.1	
24.95	brPb-670471	48.6	95.1	96.8	95.9	98.4	87.2	85.1	100	

Chromosome A3 cluster
DArT Locus	Sequence identity (%)	
	brPb-658285	brPb-658945	brPb-659037 	brPb-661767 	brPb-662944	brPb-808545	
brPb-658285	100	99.1	80.1	85.9	97.8	96.7	
brPb-658945	99.1	100	80.6	86.1	98.4	97.3	
brPb-659037 	80.1	80.6	99.3	97.1	81.4	82	
brPb-661767 	85.9	86.1	97.1	100	87.8	88.1	
brPb-662944	97.8	98.4	81.4	87.8	99.9	98.6	
brPb-808545	96.7	97.3	82	88.1	98.6	99.3	

Chromosome A6 cluster
DArT Locus	Sequence identity (%)	
	brPb-660405	brPb-662153	brPb-671060	brPb-809719	
brPb-660405	99.5	89.7	91.1	95.1	
brPb-662153	89.7	100	96.6	88.2	
brPb-671060	91.1	96.6	98.8	91.3	
brPb-809719	95.1	88.2	91.3	100	
brPb-840355	92.9	99.3	96.8	91.2	


Chromosome A9 cluster-1

DArT Locus	Sequence identity (%)	
	brPb-658120	brpb-659427	brPb-660428	brPb-662816	brPb-809030	brPb-809053	brPb-809065	brPb-839095	
brPb-658120	99.9	84	95.4	97.6	88.9	77.1	77.9	94	
brPb-659427	84	100	87.9	82.6	78.4	88.9	89	87.7	
brPb-660428	95.4	87.9	100	94.3	89.5	77.4	78.3	95.7	
brPb-662816	97.6	82.6	94.3	99.4	89.5	77.5	78.4	93.3	
brPb-809030	88.9	78.4	89.5	89.5	100	87.4	89	90.7	
brPb-809053	77.1	88.9	77.4	77.5	87.4	99.8	99.4	82.3	
brPb-809065	77.9	89	78.3	78.4	89	99.4	100	82.5	
brPb-839095	94	87.7	95.7	93.3	90.7	82.3	82.5	100	

Chromosome A9-cluster II

DArT Locus		
	brPb-661341	brPb-661438	brPb-661461	brPb-662321	brPb-670226	brPb-671041	brPb-840699	brPb-841591	
brPb-661341	99.6	99.1	99.3	99.4	98.8	99.2	98	100	
brPb-661438	99.1	100	98.7	98.7	98.6	98.3	97.4	99.1	
brPb-661461	99.3	98.7	100	99.6	97.9	98.5	97.2	99.3	
brPb-662321	99.4	98.7	99.6	100	97.9	98.5	97.2	99.3	
brPb-670226	98.8	98.6	97.9	97.9	98.8	98.8	97.1	98.8	
brPb-671041	99.2	98.3	98.5	98.5	98.8	99.3	98	99.7	
brPb-840699	98	97.4	97.2	97.2	97.1	98	98.9	98	
brPb-841591	100	99.1	99.3	99.3	98.8	99.7	98	100	

Chromosome C1

DArT Locus	Sequence identity (%)	
	brPb-838800	brPb-839122	brPb-838583	brPb-840904	brPb-839361	brPb-838928 	brPb-840955 	
brPb-838800	98.1	98.2	99.7	97.9	97.9	91.4	92.9	
brPb-839122	98.2	99.3	98.7	98.8	98.8	95.7	96	
brPb-838583	99.7	98.7	100	99.8	99.8	96.3	96.4	
brPb-840904	97.9	98.8	99.8	99.7	99.4	87.1	89.5	
brPb-839361	97.9	98.8	99.8	99.4	100	87.3	89.6	
brPb-838928 	91.4	95.7	96.3	87.1	87.3	100	97.9	
brPb-840955 	92.9	96	96.4	89.5	89.6	97.9	100	


Chromosome C3

DArT Locus	Sequence identity (%)	
	brPb-670951	brPb-662446	brPb-808216	brPb-808265	brPb-659783 	
brPb-670951	99.4	53.3	59.4	59.4	60	
brPb-662446	53.3	99.5	80.2	80	58.3	
brPb-808216	59.4	80.2	100	99	58.6	
brPb-808265	59.4	80	99	99.7	58.8	
brPb-659783 	60	58.3	58.6	58.8	100	
