# Supplementary material for: A consensus map of rapeseed (Brassica napus L.) based on diversity array technology markers: applications in genetic dissection of qualitative and quantitative traits
Source: BMC Genomics. 2013 Apr 23;14:277. doi: 10.1186/1471-2164-14-277 (PMC3641989; doi:10.1186/1471-2164-14-277)
Supplement: Additional file 5 — Comparisons between marker density and map length (in cM) in the consensus map developed from six component individual maps. [file 1471-2164-14-277-S5.rtf]

Additional file 5. Comparisons between marker density and map length (in cM) in the consensus map developed from six component individual maps
