# Supplementary material for: A consensus map of rapeseed (Brassica napus L.) based on diversity array technology markers: applications in genetic dissection of qualitative and quantitative traits
Source: BMC Genomics. 2013 Apr 23;14:277. doi: 10.1186/1471-2164-14-277 (PMC3641989; doi:10.1186/1471-2164-14-277)
Supplement: Additional file 6 — Shared markers across different mapping populations. [file 1471-2164-14-277-S6.rtf]

Additional file 6: 
(I) Shared common markers and average correlation coefficients of the marker orders for comparison among six individual maps of rapeseed populations. The total number of common markers per chromosome and population and the average correlation in marker position per overlapping linkage group with the consensus map, where the number of common markers between two linkage groups is greater than 4. Since the TN map is used as the reference map, the correlation is always 1.0.
Chromosome	AT	BS	LM	MW	SAS	TN	Consensus	
A1	15/1.00	20/-	-/-	22/1.00	40/1.00	54/1.00	110	
A2	16/-	-/-	8/0.54	11/-	-/-	58/1.00	70	
A3	25/1.00	38/-	7/0.88	28/-	41/-	103/1.00	156	
A4	23/0.99	33/-	7/-	9/0.95	26/0.96	66/1.00	97	
A5	22/0.99	42/-	2/-	16/0.99	23/0.99	43/1.00	92	
A6	21/0.99	36/-	10/0.96	28/1.00	27/0.99	63/1.00	118	
A7	5/-	-/-	-/-	23/-	16/1.00	67/1.00	81	
A8	4/-	-/-	-/-	10/-	6/-	32/1.00	35	
A9	31/1.00	12/-	3/-	11/0.99	35/-	96/1.00	145	
A10	9/0.91	4/-	-/-	23/-	24/-	52/1.00	82	
C1	6/-	8/-	-/-	13/-	6/-	42/1.00	42	
C2	10/1.00	34/-	2/-	29/-	-/-	37/1.00	58	
C3	12/1.00	-/-	5/-	6/-	-/-	65/1.00	76	
C4	4/-	-/-	-/-	3/-	2/-	41/1.00	45	
C5	2/-	-/-	-/-	3/1.00	4/1.00	22/1.00	24	
C6	-/-	-/-	-/-	2/-	11/-	42/1.00	42	
C7	6/-	14/-	-/-	11/-	-/-	21/1.00	23	
C8	2/-	-/-	-/-	-/-	-/-	31/1.00	31	
C9	4/1.00	-/-	-/-	5/-	3/-	36/1.00	39	

(II) Spearman's rank correlation (r) of the marker order for the comparison among six component maps of DH mapping populations. Consistency of marker orders was tested on the basis of shared markers. The level of significance is shown by asterisks (*): * = <0.05, ** = <0.01, **** = < 0.001. The number of shared markers (n) between component maps are also presented. Initially sub-linkage groups (when present) were aligned with the seed map of TN population in order to determine their orientation along linkage groups (A1 to A10 and C1 to C9) and then the marker orders were determined. - symbol represents to missing data. NA means correlation cannot be calculated because all the distances are the same 1/(x-mean(x)) = 1/0 in the calculation is undefined.


Linkage Group	Population	AT		BS		LM		MW		SAS		Range of shared markers	
		r	n	r	n	r	n	r	n	r	n		
A1	AT											2 to 10	
	BS	0.953**	6										
	LM	NA	3	1***	5								
	MW	0.828**	9	0.978***	10	1***	4						
	SAS	1***	4	1	2	0.698	6	1***	5				
	TN	0.866	3	0.943*	6	1***	2	0.316	4	0.926**			
A2	AT											0 to 7	
	BS		0										
	LM		0		0								
	MW		0		0	0.713	7						
	SAS		0		0		0		0				
	TN	0.791**	7		0		0		1		0		
													
A3	AT											2 to 27	
	BS	0.953**	6										
	LM	0.988***	10	0.902***	27								
	MW	1***	9	0.64	9	0.308	12						
	SAS	0.8*	6	0.178	16	0.694***	19	0.18	15				
	TN	1	2	0.571*	14	0.579**	17	0.782**	12	0.029*	18		
A4	AT											0 to 2	
	BS	-	-										
	LM	0	0	0	0								
	MW	0	0	NA	2	0	0						
	SAS	1	2	0	0	0	0	0	0				
	TN	0	0	0	0	1	2	0	0	0	0		
A5	AT											0 to 25	
	BS	0.984***	23										
	LM	0.282	25	0.973**	11								
	MW	1***	19	0.866	9	0.684	5						
	SAS	1***	20	0.832***	14	1	2	0	1				
	TN	1***	22	0.971***	12	0.975**	5	0	0	0	0		
A6	AT											3 to 20	
	BS	0.915***	10										
	LM	1***	11	0.968***	18								
	MW	0.607	8	0.922***	9	0.696	6						
	SAS	1***	10	0.972***	16	1***	20	NA	5				
	TN	0.894**	5	0.822**	8	0.991***	7	0.448	6	0.866	3		
A7	AT											0 to 13	
	BS	-	-										
	LM	NA	4	0	0								
	MW	0	0	0.067	13	0	0						
	SAS	0	0	0.988***	8	1	2	0	0				
	TN	0	0	0.949*	4	0.5	3	0.949**	5	0	0		
A8	AT											0 to 3	
	BS	0	0										
	LM	0	0	1***	3								
	MW	0	0	0	0	0	0						
	SAS	0	0	0	0	0	0	0	0				
	TN	0	0	0	0	0	0	0	0	0	0		
A9	AT											0 to 10	
	BS	NA	2										
	LM	0.975***	10	0	0								
	MW	0	5	0	0	0	0						
	SAS	0	0	0	0	0	0	0	0				
	TN	0	0	0	0	0	0	0	0	0	0		
A10	AT											0 to 10	
	BS	0	0										
	LM	1	2	1	2								
	MW	1***	5	1	3	0.992	9						
	SAS	0.775	4	1	2	0.858**	9	0.786**	10				
	TN	0	0	0.943*	6	0.971***	6	0.819**	8	0.709	7		
C1	AT												
	BS	0	0									0	
	LM	0	0	0	0								
	MW	0	0	0	0	0	0						
	SAS	0	0	0	0	0	0	0	0				
	TN	0	0	0	0	0	0	0	0	0	0		
C2	AT											0 to 15	
	BS	0.828*	6										
	LM	0	0	0	0								
	MW	0	0	0	0	0.957***	15						
	SAS	0	0	1***	5	0	0	0	0				
	TN	0.775	4	0.857	11	0	2	0	0	0.775	4		
C3	AT											0 to 5	
	BS	0	0										
	LM	1***	3	0	0								
	MW	0	0	0	0	0	0						
	SAS	-	-	-	-	-	-	-					
	TN	0.894	4	0	0	0.975	5	1	3	0	0		
C4	AT											0 to 2	
	BS	-	-										
	LM	0	0	0	0								
	MW	0	0	0	0	0	0						
	SAS	0	0	0	0	0	0	0	0				
	TN	1	2	1	2	0	0	0	0	0	0		
C5	AT											0 to 3	
	BS	0	0										
	LM	0	0	1***	3								
	MW	0	0	0	0	0	0						
	SAS	1	2	0	0	0	0	0	0				
	TN	0	0	0	0	0	0	0	0	1	2		
C6	AT											0 to 5	
	BS	-	-										
	LM	-	-	-	-								
	MW	-	-	-	-	NA	2						
	SAS	-	-	-	-	NA	2	0	0				
	TN	0.866	5	0	0	0.866	5	0	0	1	3		
C7	AT											0 to 4	
	BS	1***	4										
	LM	0	1	0	0								
	MW	0	0	0	0	0	0						
	SAS	-	-	-	-	-	-	-	-				
	TN	0	0	0	0	0	0	0	0	-	-		
C8	AT											0 to 3	
	BS	0	0										
	LM	0	0	1***	3								
	MW	0	0	0	0	0	0						
	SAS	0	0	0	0	0	0	0	0				
	TN	0	0	0	0	0	0	0	0	0	0		
C9	AT											0 to 2	
	BS	-	-										
	LM	0	0	-	-								
	MW	0	0	-	-	NA	2						
	SAS	1	2	-	-	0	0	0	0				
	TN	0	0	-	-	0	0	1	2	0	0		
