# Supplementary material for: A consensus map of rapeseed (Brassica napus L.) based on diversity array technology markers: applications in genetic dissection of qualitative and quantitative traits
Source: BMC Genomics. 2013 Apr 23;14:277. doi: 10.1186/1471-2164-14-277 (PMC3641989; doi:10.1186/1471-2164-14-277)
Supplement: Additional file 10 — Relationship between genetic map distance and physical map distance for all the 19 Brassica A and C genome chromosomes. Genetic distances are given in cM, derived from the B. napus consensus map. Map order of linkage groups was orientated according to genome scaffolds. DArT sequences which were genetically mapped on to linkage groups were only aligned against the corresponding scaffolds. DArT sequences those showed multiple sequence alignments within the linkage group (chromosome) were also included. [file 1471-2164-14-277-S10.rtf]

Additional file 10: Relationship between genetic map distance and physical map distance for all the 19 Brassica A and C genome chromosomes. Genetic distances are given in cM, derived from the B. napus consensus map. Map order of linkage groups was orientated according to genome scaffolds. DArT sequences were only aligned against the corresponding scaffold. DArT sequences those showed multiple alignments within the linkage group (chromosome) were also included.


A1

A2

A3


A4


A5


A6


A7


A8


A9


A10


C1


C2


C3


C4


C5

C6

C7


C8


C9
