# Supplementary material for: A consensus map of rapeseed (Brassica napus L.) based on diversity array technology markers: applications in genetic dissection of qualitative and quantitative traits
Source: BMC Genomics. 2013 Apr 23;14:277. doi: 10.1186/1471-2164-14-277 (PMC3641989; doi:10.1186/1471-2164-14-277)
Supplement: Supplementary file 10 [file 1471-2164-14-277-S4.rtf]

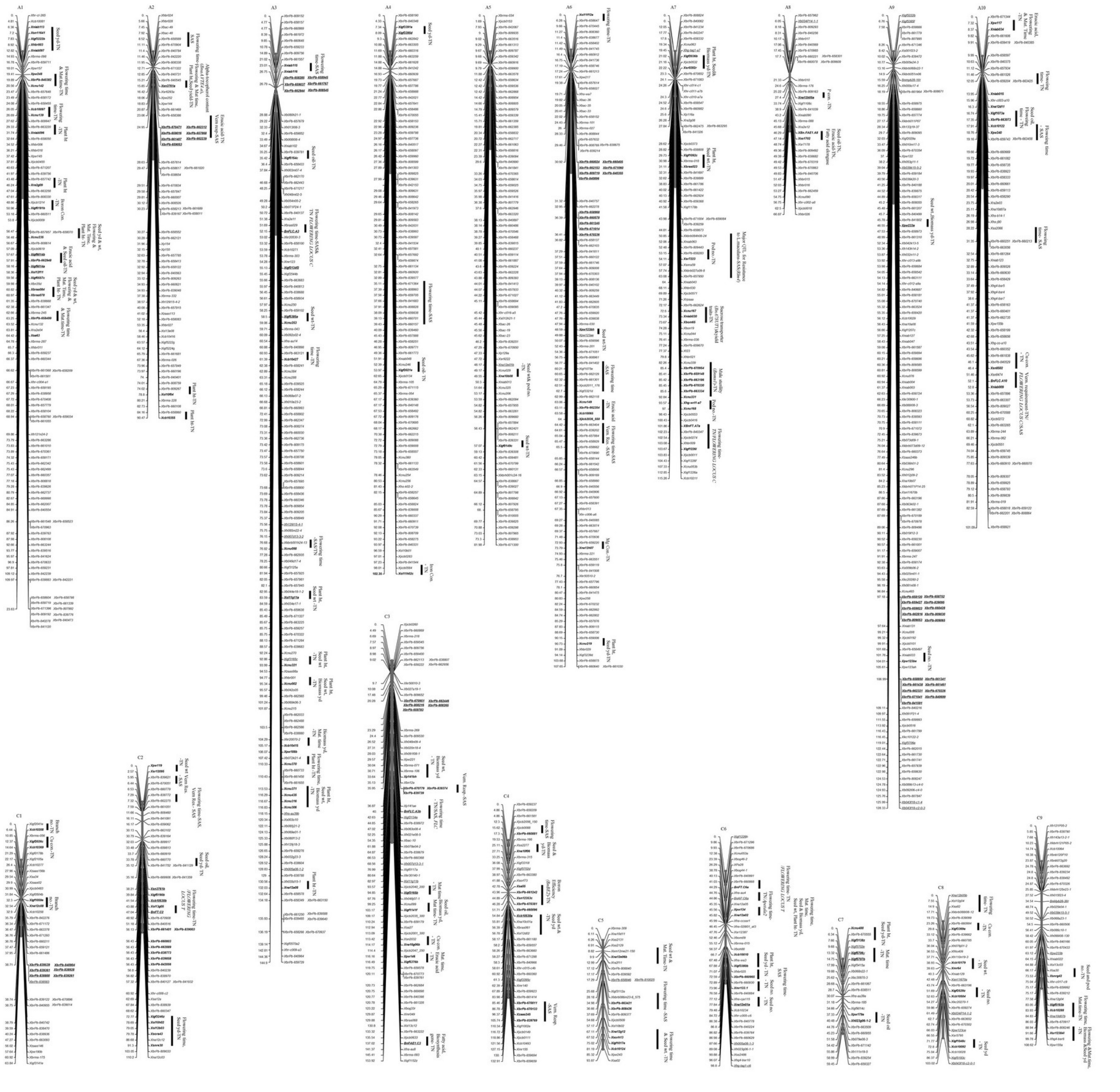
A1
A2
A3
A4
A5
A6
A7
A8
A9
A10
C1
C2
C3
C4
C5
C6
C7
C8
C9
